# Parenteral trace element provision: recent clinical research and practical conclusions

**DOI:** 10.1038/ejcn.2016.53

**Published:** 2016-04-06

**Authors:** P Stehle, B Stoffel-Wagner, K S Kuhn

**Affiliations:** 1 Department of Nutrition and Food Sciences - Nutritional Physiology, University of Bonn, Bonn, Germany; 2 Department of Clinical Chemistry and Clinical Pharmacology, University Clinic of Bonn, Bonn, Germany

## Abstract

The aim of this systematic review (PubMed, www.ncbi.nlm.nih.gov/pubmed and Cochrane, www.cochrane.org; last entry 31 December 2014) was to present data from recent clinical studies investigating parenteral trace element provision in adult patients and to draw conclusions for clinical practice. Important physiological functions in human metabolism are known for nine trace elements: selenium, zinc, copper, manganese, chromium, iron, molybdenum, iodine and fluoride. Lack of, or an insufficient supply of, these trace elements in nutrition therapy over a prolonged period is associated with trace element deprivation, which may lead to a deterioration of existing clinical symptoms and/or the development of characteristic malnutrition syndromes. Therefore, all parenteral nutrition prescriptions should include a daily dose of trace elements. To avoid trace element deprivation or imbalances, physiological doses are recommended.

## Introduction

In human physiology, inorganic elements that are found in low concentrations in body tissues and fluids are generally termed as trace elements.^1^ For nine trace elements, at least one important physiological function in human metabolism is known. These trace elements are ranked as essential micronutrients and should be regularly consumed through foods and/or supplements. Shortage of intake may result in serious metabolic impairments that may lead to stunted growth, reduced physical and mental power, higher risks of chronic diseases, prolonged recovery after illness and early death (Table 1).^2, 3, 4, 5, 6, 7, 8, 9^ Recommended Daily Allowances (RDAs) for micronutrients, including essential trace elements, are regularly published by governmental and non-governmental bodies worldwide to ensure health and well-being over a lifetime.^10, 11^ Calculations of RDAs should be based on nutrient demands (amount of nutrient required in metabolism to avoid known signs of deficiency) and should consider average absorption rates (bioavailability) from food. True metabolic demands remain unknown for most essential trace elements, even in healthy subjects. Additional information is gained from observational studies in apparently healthy populations, and the daily nutritive intake of micronutrients is used to estimate an adequate intake range. For safety reasons, upper intake levels are defined for all essential trace elements, with the exception of chromium.^10, 11^

In pathophysiological conditions, reliable information in regard to nutrient demands, including trace elements in various disease states, is generally unavailable.^12^ Critical conditions such as burns, trauma and septic shock are often associated with severe inflammation and oxidative stress, immune dysfunction and malnutrition, and metabolic requirements for vitamins and trace elements, in particular for selenium and zinc, may be increased.^13, 14, 15, 16, 17^ Whether single or multiple organ failure (for example, of the liver or kidney) leads to higher or even lower trace element requirements remains unknown.

Artificial nutrition for patients unable to orally consume sufficient food must provide all physiologically essential macronutrients and micronutrients to avoid symptoms of deficiency and to prevent further aggravation of the underlying disease. As quantitative figures for micronutrient intake during illness are unknown, the composition of enteral formulations designed for a balanced nutritional support is generally based on RDAs/adequate intakes that have been published for healthy adults.^18, 19^ As the bioavailability (plasma appearance) of nutrients parenterally administered is per definition at 100%, the dosage required to prevent deficiencies is lower compared with oral or enteral intake. The bypassing of hepatic first-pass effects may also influence trace element metabolism and distribution. Definition of adequate parenteral trace element provision can only be based on case-specific assessments of clinical/functional biomarkers and, most importantly, on safety aspects.

The goal of the present review was to compile recent clinically relevant scientific evidence, with respect to quality and quantity of parenteral trace element provision, and to draw conclusions for clinical practice when treating adult patients in need of parenteral nutrition (PN).

## Methods

### Search strategy and article selection

A systematic literature search was performed explicitly for human studies in English or German using PubMed (www.ncbi.nlm.nih.gov/pubmed) and Cochrane (www.cochrane.org) databases (last data entry: 31 December 2014). Furthermore, reference lists of reviews and original papers were reviewed. The search terms used (AND/OR) were 'trace elements, parenteral nutrition, clinical nutrition, human studies, selenium, zinc, iodine, manganese, chromium, iron, copper, molybdenum and fluoride'. Clinical studies that evaluated the effects of parenteral/intravenous trace element provision on the clinical outcome (morbidity and mortality) and biomarkers of nutritional status (for example, extracellular and intracellular concentrations) were considered as eligible.

## Results

### Selenium

Since the late 1980 s, controlled clinical studies have evaluated the effects of low-dose (<200 μg/day) parenteral selenium in surgical in-hospital and out-hospital patients supplemented either as part of a trace element preparation^20, 21, 22^ or a single selenium additive.^23, 24^ More recent studies in critically ill patients (burns, sepsis/systemic inflammatory response syndrome and bone marrow transplantation) supplied standard parenteral preparations with much higher selenium doses up to 2000 μg/day.^25, 26, 27, 28, 29^ Outside of a nutrition therapy protocol, sodium selenite was given intravenously in critical care to balance metabolic stress^30, 31, 32, 33^ in daily doses up to 4000 μg.

Recently, three systematic reviews and meta-analyses were conducted to assess the efficacy of parenteral selenium supply primarily on clinical outcomes.^34, 35, 36^ Huang *et al.*^34^ included randomised trials investigating parenteral selenium supplementation administered in addition to standard of care (12 studies with 965 critically ill patients). All-cause mortality as primary outcome was significantly reduced with a parenteral selenium provision of at least 1000 μg/day, although the studies included were observed to differ considerably in regard to methodological quality. The reported effect was linked to the duration of supplementation, whereas the dose administered (low or high) was undecisive. Alhazzani *et al.*^35^ selected for their meta-analysis randomised controlled trials (adult patients with systemic inflammatory response syndrome, sepsis, severe sepsis or septic shock; nine studies with 792 patients), comparing intravenous selenium supplementation of at least 100 μ/day with placebo or placebo with a maintenance dose of selenium (<100 μg/day). The primary outcome was either hospital mortality or 28- day mortality. Selenium was mostly given as sodium selenite and doses ranged from 474 to 2000 μg/day. Only high doses (>500 μg/day) resulted in a statistically significant reduction in mortality in the patient group compared with the control group; however, there was no statistically significant difference in the effect of selenium between low-dose and high-dose trials. There was no evidence that selenium altered the length of the intensive care unit stay or changed the incidence of nosocomial pneumonia. Manzanares *et al.*^36^ included for their systematic review and meta-analysis randomised controlled trials that supplemented trace elements and/or vitamins versus placebo (either via enteral, parenteral, or both) to critically ill adult patients (primary outcome: overall mortality). As a result of study identification and selection, 21 RCTs including 2531 patients met the inclusion criteria. There were 16 trials that evaluated selenium alone or in combination with other micronutrients in antioxidant cocktails. Selenium supplementation was associated with a trend towards a reduction in mortality. In contrast to Huang *et al.*^34^, the authors found a significant dose effect: a parenteral supply of selenium lower than 500 μg/day had no effect on mortality, whereas in four trials, using doses greater than 500 μg/day, a trend towards a lower mortality (relative risk=0.80, 95% confidence interval; *P*=0.07) was observed. Influence on the infection rate was only observed in two trials using high-dose selenium. Interestingly, when trials that did not use selenium (*n*=4) were aggregated, there was a significant reduction in mortality. A clear weakness of all three systematic reviews and meta-analyses is that the general (micro)nutrient status and/or the quantity and quality of nutrition therapy used were not considered in data analyses.

Parenteral selenium administration is shown to be safe in all studies to date, even in cases where a high initial bolus (2000 μg) was administered followed by a daily dose of 1600 μg.^28, 37^ The concentration of plasma selenium has been recommended to be monitored during PN on a regular basis, and if the concentration is found to exceed 160 μg/l over a prolonged period of time the dose should be attenuated.^38^ Particular care should be taken in patients receiving long-term PN and in those with renal failure, as selenium is primarily excreted from the kidney into urine.^37^ Therefore, routine supplementation of parenteral selenium in a dosage comparable to the quantities recommended for healthy adults may be adequate.

### Zinc

In the early days of PN, a zinc-free PN regimen was recognised to rapidly lead to zinc deficiency.^39^ In 1975, Kay and Tasman-Jones^40^ first described an acquired form of acrodermatitis enteropathica in patients receiving trace metal-free PN. Numerous cases of clinical zinc deficiency have been subsequently reported.^41^ Further evidence of the importance of zinc in pathophysiological situations via the rapid reversibility of symptoms of deficiency usually within a few days of zinc supplementation has been identified. Clinical studies primarily focusing on intravenous zinc supplementation are limited. Zinc has been primarily administered as a standard parenteral trace elements preparation.^25, 31, 42, 43^

Determination of the optimal level of zinc to be included in PN has proved difficult because patients may have a wide range of requirements, depending upon their initial zinc status and the underlying disease. To evaluate basal requirements in PN, Wolman *et al.*^44^ administered increasing doses of up to 22.8 mg zinc/day in metabolically stable patients with gastrointestinal disease. Intake of ~3.3 mg/day maintained zinc balance in the absence of gastrointestinal losses, whereas in all other patients 5.9 mg zinc/day was sufficient to counteract urinary excretion. Zinc retention was observed with an intake of 11.7 mg zinc/day. Interestingly, a positive zinc balance was necessary to achieve nitrogen retention and effective utilisation of amino acids. In catabolic patients, urinary zinc losses may be increased,^45^ whereas in burns patients zinc is additionally lost through the skin.^31^ In a retrospective observational study, an average zinc dose of 9.1 mg/day in patients with short bowel syndrome and 7.6 mg/day in patients without short bowel syndrome in long-term home PN resulted in normal serum zinc concentrations in 90% of patients from both groups.^46^ The clinical benefit of zinc has been demonstrated by applying doses of 30–35 mg zinc for 2–3 weeks in patients with burns of >20% total body surface area.^43^

Only a few cases of zinc toxicity during PN are documented in the literature and are related to accidental overprovision (10–100-fold excess).^47, 48^ Monitoring of zinc status is complicated by the lack of a single reliable indicator and by the fact that plasma and serum concentrations are maintained by homoeostatic mechanisms for several weeks, even with severe dietary zinc restriction.^37^

Because of limited studies high-dose supplementation with zinc may not be recommended. Regular infusion of approximately 5–6 mg zinc/day may be sufficient to balance metabolic needs.

### Copper

There are several reports of patients receiving long-term PN with no additional copper supplementation with clear signs of copper deficiency.^49^ In a clinical setting, the amounts of parenteral copper necessary to achieve copper balance show great variability, ranging from 0.1 mg/day in surgical patients^50^ to 3.9 mg/day in tumour-bearing patients.^51^ Doses of 1.3 mg/day have been routinely provided with standard trace element preparations and have been observed to maintain or correct plasma copper values to concentrations within or slightly above the reference range in clinical surveys of post-surgical patients^22^ and long-term PN patients.^21, 52^ In these patients, slightly enhanced plasma copper concentrations reflect the expected increase in the acute-phase protein caeruloplasmin and represent a ‘normal' serum copper in the disease state. In a recent case study, trace element deprivation including copper was reported following bariatric surgery.^53^

In a previous study, copper metabolism was analysed in 28 stable patients with gastrointestinal diseases receiving total PN. Copper balance was achieved via supplementation of 0.3 mg copper/day in patients with normal gastrointestinal functions and 0.4–0.5 mg copper/day in the presence of diarrhoea or increased fluid loss.^54^ In patients with biliary fistula and high biliary losses,^55^ an additional copper supplement may be required. During long-term home PN, copper infusion at 1 mg/day increased serum concentrations above normal ranges in almost one-quarter of patients, leading the authors to recommend a lower daily copper dosage.^46^

The provision of high doses of supplemental copper is of particular importance in patients with severe burns.^56, 57^ To compensate for considerable cutaneous losses, supplementation of 3.0–3.5 mg/day has been considered beneficial in the presence of open wounds.^58^ However, there are some concerns that excess copper resulting from trace element contamination of PN components may lead to accumulation of copper in the liver and may potentially cause liver damage.^57, 59^ Patients with significant cholestasis on total PN are most likely to accumulate more hepatic copper than patients with no or only minimal cholestasis.^60^

Therefore, it may be reasonable to limit regular copper supplementation in uncomplicated patients to approximately 0.5 mg/day.

### Manganese

Serum concentrations of manganese decrease when manganese-free PN is provided.^61^ With the aim of maintaining a physiological level of manganese, Takagi *et al.*^62^ conducted a dose-finding study in 12 stable patients receiving home PN using a broad range of daily manganese doses (0, 55, 110 and 1100 μg/day). Whole-blood manganese concentrations were within the normal range in all patients who received 55 μg/day of manganese. Using this dose, accumulation of manganese in the basal ganglia of the brain was not observed. The authors concluded that 55 μg/day of manganese represents the optimal intravenous dose to maintain manganese status during long-term PN.

In patients receiving long-term PN with daily parenteral manganese intakes as high as 500–2200 μg/day, extracellular hypermanganesemia and/or accumulation of manganese in the basal ganglia of the brain have been reported.^63, 64, 65, 66, 67, 68, 69, 70, 71, 72, 73, 74^ Recently, Abdalian *et al.*^75^ assessed the effect of manganese supplementation in 16 patients on long-term PN receiving a multi-TE preparation with a mean manganese dose of 400 μg/day. In 57% of patients, blood manganese levels were above the upper level of normal, and 81% of patients had manganese deposits in their basal ganglia. Brain deposition was found in two patients (15%) with a clinical diagnosis of Parkinson's disease. Multiple neuropsychiatric symptoms were reported and included depression (66%), lack of concentration (42%), memory disturbances (17%) and gait instability (8%). There is clear evidence of an association of increased serum or whole blood manganese in patients with cholestasis,^70, 76^ whereas in mild liver disease the effect is probably smaller in adults.^77^ These findings are of concern, particularly as patients with cholestatic liver disease are likely to retain manganese that is normally excreted via the biliary route.^57, 78^ Regular monitoring of the manganese status via erythrocyte or whole blood analyses in combination with the occasional use of magnetic resonance imaging to monitor manganese accumulation in the brain, particularly in long-term home PN patients and in patients with liver dysfunction, is recommended.^37, 78, 79^ To diminish the risk for manganese deposits in the brain, a regular dose of 50–60 μg of manganese as part of daily nutrition therapy should be given.

### Chromium

In 1977, the first clinical signs of chromium deprivation were documented in a patient receiving TPN who developed severe diabetic-like symptoms refractory to insulin supply.^80^ The addition of 200 μg of chromium to the daily PN solution for 3 weeks resulted in an alleviation of symptoms such as glucose intolerance, weight loss and neuropathy, and the patient no longer required exogenous insulin. The reversal of diabetic-like symptoms in patients receiving PN via the provision of short-term high-dose chromium has been repeatedly confirmed,^81^ and several case reports are documented.^82, 83^ A reliable biomarker of chromium status in patients receiving PN is lacking, and the early detection of chromium deficiency remains problematic in patients receiving PN. Clinical observation of the symptoms of chromium deficiency and their reversibility with chromium supplementation remains the most reliable^84^ and only generally accepted indicator of chromium deprivation.^85, 86^ In 2014, the European Food Safety Authority (EFSA) specified the available evidence to be insufficient to provide evidence of the essentiality of chromium in human metabolism in healthy and diseased adults.^87^

In a previous study, 50 μg/day of chromium was reported to balance urinary excretion.^50^ In subsequent studies in patients in intensive care, 100 μg/day was required to balance urinary losses.^88^ Using supplements, serum concentrations above the normal range were found with a chromium intake of 10 μg/day,^20^ and elevated tissue concentrations were found with an intake of 14 μg/day.^59, 83^ Many of the patients who received supplemental chromium exhibited serum, urine or tissue chromium concentrations above the normal range without clinical signs of chromium toxicity.^59, 89, 90, 91, 92^ Homoeostatic control of chromium depends on renal excretion. Therefore, chromium supplementation in patients with renal dysfunction is not recommended.^93^

On the basis of serum concentrations as a reliable biomarker, 10 μg/day of chromium should be sufficient in PN therapy.

### Iron

An insufficient iron supply over a certain period of time results in exhausted body stores. Consequently, iron deficiency anaemia is commonly observed in patients receiving long-term home PN without routine iron supplementation.^94, 95, 96^ Historical concerns that parenteral iron may be harmful when administered during infectious periods^97^ or iron overload may lead to a higher incidence of bacterial infection^98^ are not supported by contemporary evidence. Negative effects can be prevented by providing iron in relatively small doses, hence not to exceed the binding capacity of the transport protein transferrin. Thus, regular small doses may be preferable to intermittent large-dose supplementation.^99^

Safe and efficient dose findings of iron in PN vary considerably, ranging between 0.5 and 12.5 mg/day.^100^ In the study by Reimund *et al.*,^42^ a daily dose of 1 mg of iron given to 22 patients receiving home PN was sufficient to maintain serum iron concentrations.

The iron status of patients receiving PN should be continuously monitored to avoid a supraphysiological status.^101^ The analytical gold standard of iron store evaluation, bone marrow iron, is not routinely applicable; therefore, an iron overload is usually diagnosed via an abnormal liver biochemistry and a substantially elevated serum ferritin level (> 200 μg/l in men, and>150 μg/l in women). A simultaneous increase in haematocrit and transferrin saturation is also observed, with levels greater than 50% strongly suggestive of an iron overload.^101^ There is no evidence that iron stores should be supplied at concentrations above 1.5 mg/day in adults.

### Molybdenum

Cumulative urinary excretion in patients receiving molybdenum-free PN revealed negative balances of approximately 95–190 μg/day.^50, 88^ An inadequate endogenous availability may impair the function of enzymes that require molybdenum as a co-factor. Consequently, an adequate amount of molybdenum has been recommended for inclusion in PN regimens.^102^ On the contrary, only one case of molybdenum deficiency has been described during long-term PN.^103^ In this patient, treatment with ammonium molybdate (~190 μg/day) was effective in improving the clinical situation and reversing metabolic abnormalities.

A wide range of molybdenum doses have been found to be safe and efficient when administered to patients receiving home PN, with various studies reporting doses of 19 μg/day,^20^ 47.5 μg/day^42^ and 190 μg/day.^21, 103^ In early molybdenum balance studies, an intake of 19 μg/day was reported to be sufficient in maintaining physiological levels of serum molybdenum.^50^ Excessive molybdenum intake may cause significant urinary copper losses, and doses should not be increased over a long period of time.^102^ Thus, copper levels should also be monitored when administering molybdenum in patients receiving PN, and an adequate amount of copper should be also provided with molybdenum.^37^

### Iodine

Thyroidal iodine stores are suggested to be often sufficient to meet the needs of adult patients requiring TPN for at least up to 3 months.^104^ Furthermore, PN patients are frequently exposed to iodine from various exogenous sources, in particular from iodine-based skin antiseptics.^105, 106^ Non-dietary iodine provision has been considered adequate to meet basal requirements as thyroid function remains normal in the majority of long-term PN patients,^11^ and there are no reports of iodine deficiency symptoms during hospital PN.^37^ As the wide replacement of iodine-containing antiseptics with 2% chlorhexidine for skin cleansing, the risk of iodine deficiency in patients receiving PN has increased during recent years.^57^ In a recent retrospective study including 31 patients receiving long-term home PN, more than half of the patients did not receive iodine with PN.^107^ The average urinary excretion of iodine was generally low. In 3% of the patients, the serum thyroid stimulating hormone was lower than the normal reference range, whereas in 23% of the patients serum thyroid stimulating hormone levels were higher than the reference values.^107^ The findings in these two studies may support a proper iodine supplementation in patients receiving PN.

To date, there are few data available on the optimal amount of intravenous iodine. In an unpublished clinical trial, an iodine intake of 130 μg/day was associated with the maintenance or improvement of thyroid hormone concentrations (personal communication).

### Fluoride

Reliable information on adequate amounts of fluoride within a PN regimen is scarce. In an clinical trial in patients receiving postoperative PN containing 0.95 mg/day of fluoride, urine excretion was not observed to exceed 0.29–0.76 mg/day, thus confirming fluoride balance (unpublished data). Potential additional intakes of fluoride from drinking water must be considered when calculating daily supplementation.^108^ It has been recommended that fluoride levels be monitored in patients receiving long-term PN.^37, 109^ Whether parenteral fluoride may essentially contribute to the prevention or treatment of osteopenia remains unknown.^110^

## Discussion

Over the past two decades, the American Society for Parenteral and Enteral Nutrition (ASPEN),^57, 111^ European Society for Clinical Nutrition and Metabolism (ESPEN)^58, 112, 113^ and Australasian Society of Parenteral and Enteral Nutrition (AuSPEN)^114^ have published guidelines for parenteral trace element provisions (Table 2). These figures may vary when specific clinical situations such as burns are considered.

There are some discrepancies in regard to the number and optimal amounts of trace elements to be included in adults receiving PN. Whereas ESPEN has published recommendations for all of the nine trace elements known to be essential in healthy adults, ASPEN has not specified required daily intakes for iron, molybdenum, iodine and fluoride. In a recent position paper, the routine supplementation of PN with iron and iodine has been acknowledged by experts in the United States,^57^ although distinct recommendations were not provided. In 2014, AuSPEN published guidelines for the parenteral provision of all trace elements, with the exception of fluoride. Intake of iron, chromium and molybdenum was only recommended for long-term PN (Table 2). There is significant controversy surrounding the uncertainty in the actual amounts provided as part of the background trace element contamination of current PN formulations.^57^ Several earlier studies indicated that PN may be contaminated with significant quantities of other trace elements.^115, 116, 117^ To address these concerns, more recent analytical data on trace element contamination in the PN solutions used are highly desirable.

Reliable data on the metabolic demands of trace elements in different pathophysiological situations are still unavailable. Consequently, current clinical data reviewed here provide important information to confirm or to revise recommendations for parenteral provision. Indeed, most of the clinical data support the figures published by scientific societies (Table 2). Concerning parenteral fluoride supplementation, final recommendations cannot be given. In 2009, ESPEN defined an average daily fluoride intake of 0.95–1.5 mg/day for adults receiving home PN and 1 mg/day for surgical patients.^113, 114^ As suggested by the ASPEN research workshop in 2009, the recommended intake of fluoride for typical PN-fed patients should be in accordance with the recommendations for oral uptake of approximately 3–4 mg/day (Table 2).^111^ In the 2012 ASPEN position paper,^57^ supplementation of PN with fluoride was clearly stated to be potentially beneficial, but its inclusion in parenteral multi-TE preparations for use in the US is still not encouraged and further research is required.

Initiated by experimental and clinical studies reporting oxidative stress in seriously ill patients, the hypothesis that a high-dose supplementation of antioxidative nutrients including certain trace elements may improve patient outcomes has been developed within the framework of pharmaconutrition. Unfortunately, none of the recent controlled studies reviewed here were able to demonstrate any clinical benefits, and consequently guidelines for PN should still adhere to a recommendation of physiological doses of trace elements.

As previously outlined, there is convincing evidence that the nine trace elements known to be essential in healthy adults are equally important in disease states. Consequently, these trace elements should be a basic component of any long-term artificial nutrition regimen. Multi-trace element preparations are presently available and provide physiological doses of trace elements that adhere with current recommendations. In critically ill situations, requirements for single trace elements may be considerably increased, thus necessitating specific treatment with mono-element solutions.

Blood trace element analysis should be periodically performed to control dosage. Physical examinations including evaluation for trace element deficiency or symptoms of toxicity are recommended. Care should be taken to control contamination of trace elements from medical material such as gloves and tubes to avoid an overdose.

## Figures and Tables

**Table 1:** Essential trace elements: physiological functions and symptoms of deficiency in humans^2, 3, 4, 5, 6, 7, 8, 9^

*TE*	*Physiological function(s)*	*Symptoms of dietary deficiency*	*Symptoms in patients on long-term PN without added TE or inadequate provision of TE*
Selenium (Se)	Component of >25 selenoproteins representing the functional form of selenium and involved in redox signalling, the antioxidant defence system (glutathione peroxidase), thyroid hormone metabolism and immune response	Cardiomyopathy, chronic osteoarthritis, poor immune function, cognitive decline, increased risk of autoimmune thyroid disease, impaired reproductive capacity In the acute-phase of critical illness: oxidative stress, infectious complications, worsening organ failure, higher mortality rates	Nail and hair changes in children, skeletal muscle myopathy, cardiomyopathy
Zinc (Zn)	As a component of >300 zinc enzymes: essential for health and well-being, has an important role in wound healing, required for the structural integrity of proteins regulating gene expression and nuclear binding proteins acting as transcription factors	Growth retardation, delay in sexual maturation, diarrhoea, increased susceptibility to infections, dermatitis, the appearance of behavioural change, alopecia, delayed wound healing, impaired resistance to infection, reduced growth rate	Eczematous rash, nail changes, alopecia, mental apathy and depression, visual dysfunction, impaired immune function
Copper (Cu)	Component of copper metalloenzymes (cuproenzymes) required for normal function of the haematologic, vascular, skeletal, antioxidant and neurologic systems	Anaemia, leukopenia, bone abnormalities, decreased pigmentation of skin and hair, neurological derangements	Anaemia, pancytopenia, neutropenia
Manganese (Mn)	Cofactor for the activity of many metalloenzymes involved in antioxidant protection and amino acid, lipid, protein, and carbohydrate metabolism	A specific deficiency syndrome has not been described in humans.	No cases in adults, one isolated case of a paediatric patient with manganese deficiency (short stature, low serum levels, depletion of bone manganese levels)
Chromium (Cr)	Required for normal glucose tolerance and lipid metabolism (promotion of insulin action in peripheral tissues)	Postulated as a contributing factor to the development of type II diabetes.	Syndrome of glucose intolerance similar to diabetes
Iron (Fe)	Major component of several important classes of functional proteins (haeme-proteins, enzymes, storage and transport proteins) involved in O_2_ and electron transport	Anaemia, reduced resistance to infection; in clinical setting: adverse effects on outcome parameters	Iron deficiency anaemia
Molybdenum (Mo)	Part of the molybdenum co-factor (molybdopterin) of several flavo- and haeme enzymes involved in oxidation–reduction reactions, amino acid and purine metabolism	A dietary deficiency of molybdenum has never been reported in humans	One isolated case reported (progressive tachycardia, tachypnoea, neurological and visual changes, and coma), associated with increased excretion of sulphite, hypoxanthine and xanthine, and reduced excretion of uric acid and sulphate.
Iodine (I)	Major component of the thyroid hormones, triiodothyronine (T3) and thyroxine (T4), modulating resting energy expenditure and being important in growth and development	Iodine deficiency disorders: goitre, cognitive defects (cretinism), teratogenic effects	Not yet reported in patients on PN due to the widespread use of iodine-containing antiseptics.
Fluoride (F)	Protection against dental caries and osteoporosis	No specific deficiency syndrome	

**Table 2:** International recommendations for parenteral trace element provision in adult patients

	*ASPEN 2004*^111^	*ESPEN 2009*^112, 113^	*ASPEN 2012*^57^	*AuSPEN 2014*^114^
Selenium (Se), μg/day	20–60	20–72	60–100	60–100
Zinc (Zn), mg/day	2.5–5	2.5–6.5	3–4	3.2–6.5
Copper (Cu), mg/day	0.3–0.5	0.3–1.5	0.3–0.5	0.3–0.5
Manganese (Mn), mg/day	0.06–0.1	0.165–0.3	0.055	0.055
Chromium (Cr), μg/day	10–15	10–15	⩽1^a^	10–15
Iron (Fe), mg/day	NS	1.0–1.2	NS	1.1^b^
Molybdenum (Mo), μg/day	NS	19.5—25.5	NS	19^b^
Iodine (I), μg/day	NS	1.3–130	NS	130^b^
Fluoride (F), mg/day	NS	1	NS	NS

Abbreviation: NS, not specified.

a In addition to the current multiple TE preparations containing 10–15 μg/day.

b For longer-term PN only.
